# Mechanisms Underlying Footshock and Psychological Stress-Induced Abrupt Awakening From Posttraumatic “Nightmares”

**DOI:** 10.1093/ijnp/pyv113

**Published:** 2015-11-21

**Authors:** Bin Yu, Su-Ying Cui, Xue-Qiong Zhang, Xiang-Yu Cui, Sheng-Jie Li, Zhao-Fu Sheng, Qing Cao, Yuan-Li Huang, Ya-Ping Xu, Zhi-Ge Lin, Guang Yang, Jin-Zhi Song, Hui Ding, Yong-He Zhang

**Affiliations:** Department of Pharmacology, Peking University, School of Basic Medical Science, 38 Xueyuan Road, Beijing, 100191, China (Ms Yu, Zhang, Li, Cao, Song, and Ding, Drs S-Y Cui, X-Y Cui, and Y-H Zhang, and Mr Sheng, Huang, Xu, Lin and Yang).

**Keywords:** 5-HT, female rats, NE, Orexin, startled awakening

## Abstract

**Background:**

Posttraumatic nightmares are a highly prevalent and distressing symptom of posttraumatic stress disorder (PTSD), but have been the subject of limited phenomenological investigations.

**Methods:**

We utilized a communication box to establish PTSD symptoms in rats through exposure to footshock stress (FS) and psychological stress (PS). The immunohistochemical test and high-performance liquid chromatography with electrochemical detection were used to detect the activity and monoamine levels in the rats’ arousal systems.

**Results:**

Twenty-one days after traumatic stress, 14.17% of FS and 12.5% of PS rats exhibited startled awakening, and the same rats showed hyperfunction of the locus coeruleus/noradrenergic system and hypofunction of the perifornical nucleus/orexinergic system. Changes in serotonin levels in the dorsal raphe nucleus showed opposite trends in the FS and PS rats that were startled awake. No differences were found in other sleep/arousal systems.

**Conclusion:**

These results suggest that different clinically therapeutic strategies should be considered to treat different trauma-induced posttraumatic nightmares.

## Introduction

Nightmares are frequently associated with posttraumatic stress disorder (PTSD) and clinically relevant in today’s world of violence. Interrupted nightmares (i.e. abrupt awakening from nightmares) may have a causal or sustaining effect on sleep disturbances (Woodward et al., 2000; DeViva et al., 2004). This will consequently increase the dreamer’s distress and reinforce the trauma-related memory. As a result, interrupted nightmares can lead to the maintenance or even exacerbation of the severity of PTSD symptoms (Phelps et al., 2008). However, the mechanisms that underlie abrupt awakening from posttraumatic nightmares remain unclear, and such nightmares are difficult to treat, with few pharmacological options.

Generally, awakening is regulated by an ascending arousal system that contains the following key components: cholinergic (Ach) cell groups in the upper pons (pedunculopontine [PPT] and laterodorsal tegmental [LDT] nuclei); norepinephrine (NE) cell groups in the locus coeruleus (LC); and serotonin (5-hydroxytryptamine [5-HT]) cell groups in the dorsal raphe nucleus (DRN) and median raphe nucleus (MnR). Perifornical (PeF) orexin neurons promote the rapid sleep-wake transition and state maintenance (Peplow, 2013). In the present study, we found some rats that were exposed to footshock stress or psychological stress 21 days previously exhibited startled awakening (see Supplementary Videos S1, S2, and S3). We hypothesized that this “startled awake” behavior might mimic the phenomenon of posttraumatic nightmares in PTSD.

PTSD can be triggered not only in people who personally experienced traumatic events but also in those who witnessed them (Galea et al., 2002; American Psychiatry Association, 2013). Here we used a communication box to establish PTSD symptoms in rats through exposure to footshock stress (FS) or psychological stress (PS). These two models simulate a person who experiences or witnesses a traumatic event, respectively (Watanabe et al., 1990). Considering the fact that the incidence of posttraumatic nightmares is higher in women (Levin and Nielsen, 2007), we used female Sprague-Dawley rats in the present study to explore the mechanisms that underlie startled awakening by detecting the activity and levels of monoamines in arousal systems.

## Methods

### Animals

Female Sprague-Dawley rats (250–270g, purchased from the Animal Center of Peking University) were individually housed in plastic cages and maintained under an artificial 12h light/dark cycle (lights on 08:00 20:00 hours) at 23±1°C and 50±10% humidity. The rats had *ad libitum* access to food and water. The rats were randomly divided into three groups: control group (exposure to communication box without any stress); FS group (exposure to footshock stress); and PS group (exposure to psychological stress). All of the experiments were conducted in accordance with the European Communities Council Directive (2010/63/EU) for the use of experiment animals and approved by the Peking University Committee on Animal Care and Use.

### Communication Box

The communication box (64cm × 64cm × 8cm) was equipped with a floor grid that was composed of stainless steel rods placed 1cm apart. The box consisted of 16 compartments (16cm × 16cm × 8cm) that were separated by transparent plastic plates. Rats in eight compartments were exposed to an electrified grid, through which electric shocks were delivered. The other eight compartments had plastic plates (8cm × 8cm) that were placed on the grid to prevent the rats from receiving the electric shocks. The FS group was placed directly on the electric grid floor. The PS group was placed in the compartments with plastic plates so that they could observe/witness the rats that were exposed to FS stress, thus inducing psychological stress (Supplementary Figure S1A).

### Experimental Procedure

The rats were introduced to the communication box to receive FS or PS in the proestrous phase. It has been reported that rats can develop stable symptoms of PTSD, including strong emotional memory of traumatic events, 21 days after stress (Dudai, 2004; Berardi et al., 2014). Thus, to evoke the trauma-related memory, 21 days after stress exposure the rats were returned to the communication box in the absence of electric shocks. Freezing behavior was recorded in the meantime. Rats were then moved to individual sleep boxes in a noise-attenuated environment. We recorded the rats’ sleep for 6h and monitored whether they exhibited startled awakening (Supplementary Figure S1B). The rats were sacrificed immediately after they were startled awake (3–6h after sleep onset) and perfused for immunohistochemistry, or the areas of interest were dissected for monoamine detection. The FS control (FSC) and PS control (PSC) groups consisted of rats that did not present startled awakening and were sacrificed immediately after normal awakening (at the end of the 6h sleep recording). Rats in the control group were sacrificed immediately after normal awakening 3–6h after sleep onset, at the end of sleep recording. Fos expression and monoamine levels were similar in the two subgroups, and the data of the control group were pooled.

### Histological and Immunohistochemical Procedures

Tissue preparation and immunohistochemical staining were performed according to previously described methods (Wang et al., 2015). Under deep anesthesia with chloral hydrate (300mg/kg, i.p.), the rats were first perfused with 500ml of 4% paraformaldehyde. Whole brains were postfixed in the same fixative at 4°C for 24h and then immersed in 30% sucrose at 4°C for cryoprotection. The brains were then rapidly frozen on dry ice and cut into 20 μm coronal sections with a cryostat (Leica CM1850, Leica Microsystems UK). The sections were stored at -20°C until staining.

For the immunohistochemical experiments that involved nuclei that regulate awakening (PeF, bregma -3.14mm; LC, bregma -9.68mm; DRN/MnR/PPT, bregma -7.80mm; LDT, bregma -8.80mm; Paxinos and Watson, 1998), we used antibodies that specifically detect tyrosine hydroxylase (TH) for the LC (1:500; sc-14007, Santa Cruz Biotechnology), tryptophan hydroxylase (TrpOH) for the DRN and MnR (1:1000; 9260-2505, Biogenesis), choline acetylase for the LDT and PPT (1:300; ab18736, Abcam), and orexin-A for the PeF (1:50; sc-8070, Santa Cruz Biotechnology). We then labeled the activity of awakening-related nuclei by marking the expression of Fos (1:100; sc-52, Santa Cruz Biotechnology). Fos is often considered an index of neuronal activation. Neurotransmitter-synthesizing enzymes in relevant awakening-related nuclei and Fos protein expression were determined in sequential sections using diaminobenzidine (DAB) and DAB with nickel enhancement as chromogens, respectively. Cytoplasms that contained neurotransmitter-synthesizing enzymes were stained brown, and Fos-immunoreactive neurons were stained black-purple.

### Fos Protein Expression Analysis

The cell counting details were described previously (Wang et al., 2015). Photomicrographs of various brain areas from anatomically matched sections were captured using a charge-coupled device camera (Leica DC 300) and light microscope with a 10× objective for most regions and a 20× objective for the LC (Leica DMR; Leica Microsystems). For double-immunostained sections of arousal regulation nuclei, Fos-positive ratios were calculated. Immunoreactive nuclei were counted bilaterally using at least two serial sections for each area. The data were then averaged to produce group means.

### Transmitter Analysis by HPLC-ECD

The rats were sacrificed immediately after startled awakening or normal awakening in the stress and control groups, respectively. The brains were extracted, and 12-gauge tissue punches of the PeF (bilaterally), LC (bilaterally), ventrolateral preoptic nucleus (bilaterally) and DRN were obtained. Details of the neurotransmitter analysis procedure were described previously (Zhang et al., 2014). High-performance liquid chromatography (HPLC) with electrochemical detection was used to determine monoamine neurotransmitter levels under the following conditions: flow rate (0.60ml/min), temperature (40°C), column (Shiseido Capcell Pak C18 MG F90816 column; 3.0mm inner diameter, 75mm length, 3 μm pore size), injection volume (20 μl partial loop), mobile phase (0.1M NaH_2_PO_4_, 0.85mM octane sulfonic natrium (OSA), 0.05mM Na_2_EDTA (EDTA, eathylene diamine tetraacetic acid), 11% CH_3_OH, pH 3.25 with H_3_PO_4_), detector, and conditions (analytical cell: 5011A, the electrochemical detector was set with an oxidizing potential of +200 mV and reducing potential of -175mV; guard cell: 5020, the potential of guard cell was set at +250 mV).

### Statistical Analysis

The data are expressed as mean ± standard error of the mean. The data were analyzed using one-way analysis of variance (ANOVA) followed by the Student-Newman-Keuls test for multiple comparisons. Values of *p* < 0.05 were considered statistically significant.

## Results

### Footshock and Psychological Stress Caused Some Startled Awakening

During sleep monitoring, 14.17% (17/120) of the FS rats (Supplementary Video S2) and 12.5% (10/80) of the PS rats (Supplementary Video S3) exhibited startled awakening: that is, they suddenly woke up from undisturbed sleep with jumping behavior. Rats that awoke normally did not present this extreme fashion of awakening (Supplementary Video S1). Furthermore, both FS and PS rats that exhibited startled awakening also presented a long freezing time, compared with the control group, when they were returned to the communication box 21 days after traumatic stress (*F*_4, 255_ = 48.028, *p* < 0.01; Supplementary Figure S1E), indicating that they had formed strong fear memory of the traumatic event, a core symptom of PTSD (Siegmund and Wotjak, 2006).

### Orexin Neuron Activity Decreased in the Perifornical Nucleus in Rats That Exhibited Startled Awakening

**Figure 1.**
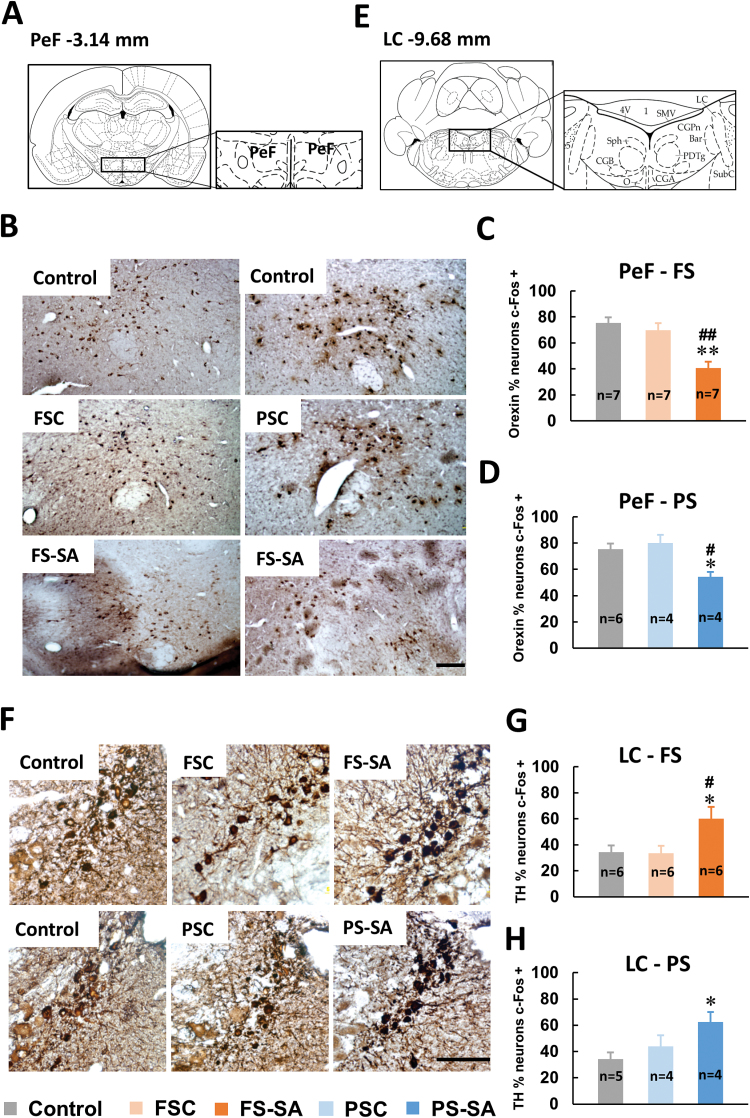
Fos expression in orexin A-immunostained neurons in the perifornical nucleus (PeF) and tyrosine hydroxylase (TH)-immunostained neurons in the locus coeruleus (LC). (A) Illustrations of brain sections of the PeF based on Paxinos and Watson (1998). (B) Photomicrographs of Fos expression in orexin A-immunostained neurons in the PeF. Scale bars = 100 μm. (C, D) Compared with normal-awakening rats, footshock stress (FS) and psychological stress (PS) rats that exhibited startled awakening (FS-SA and PS-SA, respectively) both had decreased Fos expression ratios in Orexin-A-positive neurons in the PeF. The data are expressed as mean ± standard error of the mean. (E) Illustrations of brain sections of the LC. (F) Photomicrographs of Fos expression in TH-immunostained neurons in the LC. Scale bars = 100 μm. (G, H) Compared with normal-awakening rats, the FS-SA and PS-SA groups both increased the Fos expression ratio in TH-positive neurons in the LC. *p < 0.05, **p < 0.01, different from control group; ^#^ *p* < 0.05, ^##^ *p* < 0.01, different from the FS control (FSC) and PS control (PSC) groups (Student-Newman-Keuls test).

The PeF is enriched in orexin-containing neurons. Orexins are hypothalamic neuropeptides that exist in two forms: orexin-A and orexin-B (Sakurai, 2007). We chose an antibody that targets orexin-A. In the PeF, the Fos expression ratio in orexin-A-positive neurons was significantly reduced in both the FS-startled awakening (FS-SA; *F*_2, 18_ = 14.038, *p* < 0.01; Figure 1C) and PS-startled awakening (PS-SA; *F*_2, 11_ = 6.800, *p* = 0.012; Figure 1D) groups.

### Norepinephrine Neuron Activity Increased in the Locus Coeruleus in Rats That Exhibited Startled Awakening

The LC is an arousal-promoting nucleus where NE neurons are clustered. Fos- and TH-immunostained neurons in brain sections of the LC are shown in Figure 1F. Compared with the control groups, the Fos expression ratio in TH-positive neurons was significantly increased in the LC in both the FS-SA group (*F*_2, 15_ = 4.772, *p* = 0.025; Figure 1G) and PS-SA group (*F*_2, 10_ = 5.007, *p* = 0.031; Figure 1H).

### Monoamine Changes in Awakening-Related Nuclei in Rats That Exhibited Startled Awakening

**Figure 2.**
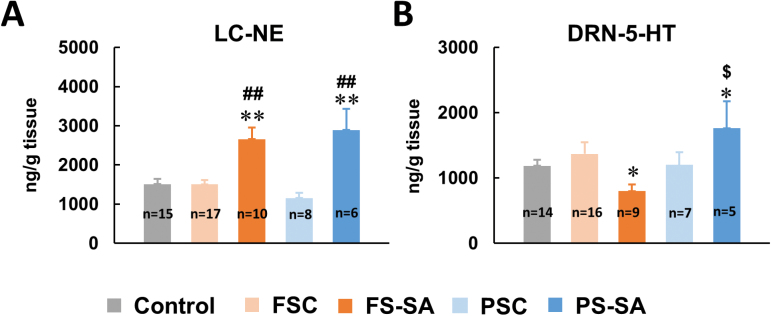
Monoamine levels in awakening-related nuclei. (A) Norepinephrine levels significantly increased in the locus coeruleus (LC) in both the footshock stress startled awakening (FS-SA) and psychological stress startled awakening (PS-SA) groups. (B) 5-hydroxytryptamine (5-HT) levels significantly decreased in the dorsal raphe nuclei (DRN) in FS-SA rats. 5-HT levels significantly increased in PS-SA rats. The data are expressed as mean ± standard error of the mean. *p < 0.05, **p < 0.01, different from control group; ^##^ *p* < 0.01, different from the FS control (FSC) and PS control (PSC) groups; ^$^ *p* < 0.05, different from the FS-SA group (Student-Newman-Keuls test).

To elucidate the monoaminergic basis of startled awakening, we detected monoamine levels in awakening-related nuclei. HPLC revealed that NE levels significantly increased in the LC in both the FS-SA (*p* < 0.01 vs FSC; Figure 2A) and PS-SA (*p* < 0.01 vs PSC; Figure 2A) groups. Serotonin levels increased in the DRN in the PS-SA group and decreased in the FS-SA group (*F*_4, 46_ = 7.839, *p* < 0.01; Figure 2B). No monoamine changes were observed in other sleep- or wake-related nuclei (Supplementary Table S1).

## Discussion

The DSM-5 specifically delineates “traumatic nightmares” among the intrusion symptoms of PTSD (American Psychiatric Association, 2013). Disturbed dreaming has been consistently found to be an integral feature of the re-experiencing/intrusion symptom cluster of PTSD across a broad range of traumatic events (Mellman and Pigeon, 2005). Sleep disturbances that are accompanied by posttraumatic nightmares may contribute to the pathophysiology of maladaptive stress responses. In the present study, we used the “startled awake” animal model to uncover awakening-related neurocircuitry that underlies “interrupted nightmares.” The present results indicated that PeF orexinergic, LC noradrenergic, and DRN serotoninergic systems might be involved in the regulation of abrupt awakening. However, the PPT/LDT Ach system did not present significant changes among groups (Supplementary Figure S2).

Orexin plays a role in the regulation of wakefulness through interactions with efferent systems that mediate arousal and energy hemostasis (Sakurai, 2007). The activation of orexin neurons may contribute to the promotion or maintenance of wakefulness. Conversely, the relative inactivity of orexin neurons may allow the expression of sleep (Estabrooke et al., 2001). Figure 1 shows that orexin neuron activity was very high in normal-awakening rats, indicating a readiness for the transition from being asleep to awake. Activity was relatively low in the FS and PS groups that exhibited startled awakening, indicating that orexin neurons were not fully prepared for awakening at that moment. Clinical analyses showed that cerebrospinal fluid and plasma orexin-A concentrations were signiﬁcantly lower in patients with PTSD compared with healthy control subjects (Strawn et al., 2010), which was consistent with the present results. Our findings suggest that low orexin-A activity might be related to the symptom of abrupt awakening.

The present data also suggest that hyperfunction of the LC noradrenergic system may be an important and common factor in causing FS and PS rats to be startled awake. These findings are consistent with previous clinical evidence, in which higher NE concentrations were found in the cerebrospinal fluid in patients with PTSD and associated with a greater severity of PTSD symptoms (Geracioti et al., 2001). Increases in NE in the central nervous system are associated with hyperarousal and re-experiencing symptoms, emotional and physiological reactions to traumatic cues, and traumatic memory retrieval and reconsolidation (Sara, 2009; Pitman et al., 2012). In the case of being startled awake, the enhancement of the LC noradrenergic system activity elicited a hyperarousal state, thus increasing attention and enhancing the perception of traumatic memories and fear emotions during nightmares. This may explain why the disturbed nightmares would maintain or exacerbate PTSD symptom severity. To date, the α-1 adrenergic receptor antagonist prazosin is recommended for the treatment of PTSD-related nightmares (Green, 2014).

We also found that the FS and PS groups that exhibited startled awakening presented opposite modifications in 5-HT levels in the DRN, indicating that the two stressors had different effects on 5-HT transmission. The brain’s 5-HT system is involved in the regulation of stress and anxiety and has also been linked to the neurobiology of PTSD. According to Harvey and colleagues (2004), 5-HT is both anxiogenic and anxiolytic and has a bidirectional role in the development of PTSD. We currently cannot explain why the different types of stress exposure that caused the rats to be startled awake were accompanied by opposite changes in 5-HT. The results showed that Fos expression of TrpOH positive neurons (Supplementary Figure S2) and the 5-HIAA/5-HT ratio in the DRN (Supplementary Figure S4) didn’t change significantly in the FS-SA and PS-SA groups. Whether other factors, such as the TrpOH-2 level, are responsible for the opposite changes of 5-HT in the FS-SA and PS-SA groups needs further investigation. Selective serotonin reuptake inhibitors (SSRIs), such as paroxetine and sertraline, are approved by the US Food and Drug Administration as first-line treatments for PTSD, but their therapeutic efficacy for nightmares has been inconsistent across clinical trials (van Liempt et al., 2006; Hasler and Germain, 2009). Based on the present findings, individual differences in the therapeutic effects of SSRIs might be attributable to different levels of changes in 5-HT that are caused by different types of stress. Different therapeutic strategies that act by increasing 5-HT levels should be reconsidered for the treatment of different trauma-induced posttraumatic nightmares.

Nevertheless, there are some limitations to the present study. First, whether all FS and PS rats, including FS-SA and PS-SA rats, would develop PTSD remains to be addressed. Second, the study did not explore how the remote traumatic memories were retrieved or the negative emotions were evoked while the startled awakening happened. Finally, the HPLC data represent neurotransmitter tissue levels, but functional modifications in monoaminergic neurotransmission were not investigated. To make up these limitations, further study is necessary.

Overall, the present study identified some of the mechanisms that underlie FS- and PS-induced abrupt awakening. We also found differences in the neurobiological basis of startled awakening between two different types of stress. The startled awake phenomenon provides a rational approach to explore the mechanisms of the sleep-wake regulation of interrupted nightmares, which may provide new perspective for the treatment of PTSD.

## Supplementary Material

For supplementary material accompanying this paper, visit http://www.ijnp.oxfordjournals.org/

## Statement of Interest

None.

## Supplementary Material

Supplementary Videos S1, S2, and S3
