# Supplementary material for: Mechanisms Underlying Footshock and Psychological Stress-Induced Abrupt Awakening From Posttraumatic “Nightmares”
Source: Int J Neuropsychopharmacol. 2015 Nov 21;19(4):pyv113. doi: 10.1093/ijnp/pyv113 (PMC4851262; doi:10.1093/ijnp/pyv113)
Supplement: Supplementary Videos S1, S2, and S3 [file Supplementary_materials_Yu_et_al.doc]

Supplementary Materials for

**Mechanisms underlying footshock and psychological stress-induced abrupt awakening from posttraumatic “nightmares”**

Bin Yu, BS; Su-Ying Cui, PhD; Xue-Qiong Zhang, BS; Xiang-Yu Cui, PhD; Sheng-Jie Li, BS; Zhao-Fu Sheng, BS; Qing Cao, BS; Yuan-Li Huang, BS; Ya-Ping Xu, BS; Zhi-Ge Lin, BS; Guang Yang, BS; Jin-Zhi Song, MS; Hui Ding, MS; Yong-He Zhang, PhD.

Department of pharmacology, Peking University, School of Basic Medical Science, 38 Xueyuan Road, Beijing, 100191, China.

**Correspondence Author:** Yong-He Zhang, Department of Pharmacology, Peking University, School of Basic Medical Science, 38 Xueyuan Road, Beijing 100191, China. E-mail: zhyh@hsc.pku.edu.cn. Fax: +86-10-82801112.

**This PDF file includes:**

Supplemental Methods

Supplemental Results

Figures S1 to S4

Table S1-S2

Captions for Videos S1 to S3

**Other Supplementary Materials for this manuscript includes the following:**

Videos S1 to S3

**Supplemental Methods**

**Estrous cycle determination**

Preliminary experiments indicated that the incidence of startled awakening was highest when rats were exposed to stress in the proestrus phase (Table S2). Thus, stress exposure and retrieval sessions were performed in rats in the proestrus phase. Vaginal swabs and cycle phase assessment were conducted between 9:00 A.M. and 10:00 A.M. for at least 5 consecutive days prior to foot-shock and psychological stress. Briefly, vaginal secretion was collected by inserting the tip of a plastic pipette filled with 10 μl of physiological saline into the rat vagina. One drop of the smear was collected with a clean tip from each rat. The vaginal fluid was then placed on glass slides. The samples were examined under a light microscope fitted with 8× and 15× objective lenses. The estrous cycle consists of 4 different phases: diestrus, proestrus, estrus and metestrus.

**FS and PS animal models establishment**

Modeling experiment was conducted between 9:00 A.M. and 11:00 A.M. in a separate quiet room, rats in proestrus phase were introduced into the communication box. FS rats received 50 shocks in 50 min. (Fig. S1C). Shock intensity was 2 mA for 10 trails, 2.5 mA for 10 trails, 3 mA for 10 trails, 3.5 mA for 10 trails, 4 mA for 10 trails, and shock intensity increased every 10 min. Each shock was 1s long and consisted of 0.01 s shocks separated by 0.02 s breaks.

**Traumatic memory retrieval**

Twenty-one days after stress exposure, to evoke the trauma related memory, rats were returned to communication box in the absence of electric shock for 10 min. Freezing behavior was recorded in the meantime. Freezing was defined as lack of movement, with the exception of those related to respiration. Measurements of freezing behavior were taken from videotaped recording by an observer blind to the experimental condition of each rat.

**Supplemental Results**

**The Fos expression in serotonergic neurons in the DRN and MnR**

We also detected the Fos expression in TrpOH immunostained neurons in the DRN and MnR. Compared with control group and FSC/PSC group, the c-Fos positive ratio in 5-HT neurons in the DRN revealed no significant difference in both FS-SA (*F*_2, 18_ = 2.779, *p* = 0.089; Fig. S2C) and PS-SA (*F*_2, 11_ = 0.668, *p* = 0.532; Fig. S2D) groups, Fos expression positive ratio in 5-HT neurons in the MnR also revealed no difference in both FS-SA (*F*_2, 18_ = 0.636, *p* = 0.541; Fig. S2E) and PS-SA (*F*_2, 11_ = 0.650, *p* = 0.537; Fig. S2F) groups.

**The Fos expression in cholinergic neurons in the PPT and LDT**

The cholinergic (Ach) cell groups in the upper pons, the PPT and LDT, play an important role in sleep-wake regulation, especially for rapid eye movement sleep. We also detected the c-Fos expression in Ach neurons in the PPT and LDT. Compared to control group and FSC/PSC group, Fos expression positive ratio in Ach neuron in the PPT revealed no difference in both FS-SA (*F*_2, 18_ = 0.425, *p* = 0.660; Fig. S2K) and PS-SA (*F*_2, 11_ = 2.750, *p* = 0.108; Fig. S2L) groups. Fos expression positive ratio in Ach neurons in the LDT revealed no significant difference in both FS-SA (*F*_2, 18_ = 0.144, *p* = 0.867; Fig. S2M) and PS-SA (*F*_2, 11_ = 0.711, *p* = 0.512; Fig. S2N).

**Monoamine changes in ventrolateral preoptic nucleus in rats that were exposed to footshock stress and psychological stress and exhibited startled awakening**

We detected monoamine levels in ventrolateral preoptic nucleus (VLPO) which is a sleep-promoting nucleus (Peplow, 2013). HPLC revealed that NE levels significantly increased in the VLPO in both the FS-SA (*p* < 0.01 vs FSC) and PS-SA (*p* < 0.01 vs PSC; Fig. S3A) groups. Compared to FS-SA group, serotonin level increased in the VLPO in the PS-SA group (*p* < 0.05; Fig. S3B).

**Supplemental Figures**


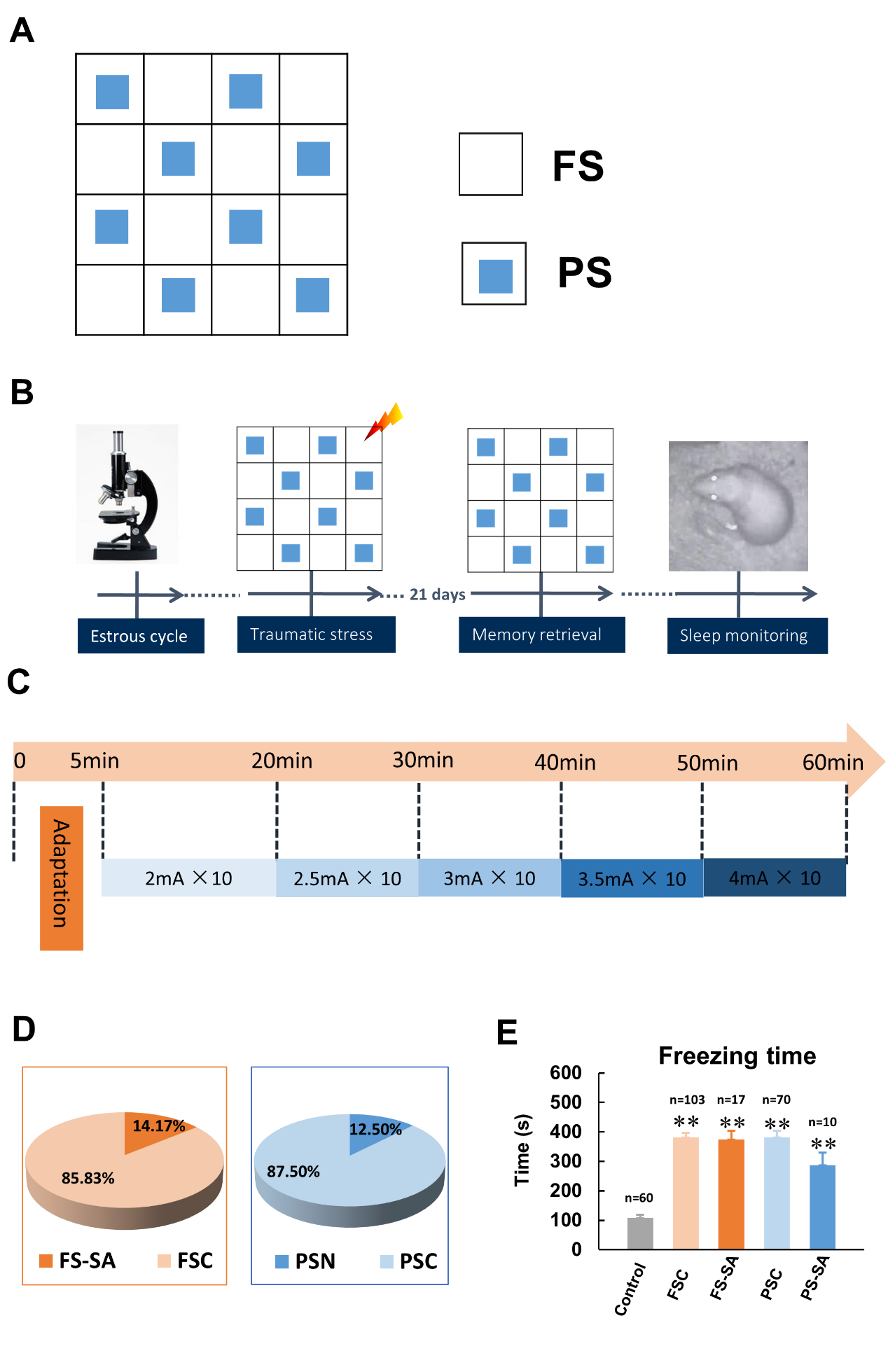


**Figure S1. The schematic diagram of communication box and experimental procedure. A.** The schematic diagram of communication box. Rats in the blank lattices exposed to the electrified grid directly, they received foot-shock stress (FS). While rats in the lattices with blue board could avoid foot-shock, they exposed psychological stress (PS) through receiving the visual, olfactory and auditory stimuli from physiological stress. **B.** Experimental procedure. Firstly, the estrous cycle of rats were determined based on the characteristics of rats’ vaginal smear. Then rats were introduced to the communication box to receive FS and PS in the proestrus phase. Twenty-one days after stress exposure, to evoke the trauma-related memory, the rats were returned to the communication box in the absence of electric shocks. Rats were then moved to individual sleep boxes in a noise-attenuated environment. We recorded rats sleep for 6 hours and monitored whether they exhibited startled awakening. **C.** Procedure of foot-shock and detailed electric parameters. FS rats received 50 shocks in 50 min. Shock intensity was 2 mA for 10 trails, 2.5 mA for 10 trails, 3 mA for 10 trails, 3.5 mA for 10 trails, 4 mA for 10 trails, and shock intensity increased every 10 min. each shock was 1s long and consisted of 0.01s shocks separated by 0.02s breaks. **D.** The incidence of FS and PS-induced startled awakening. **E.** When rats re-exposed to the communication box, freezing time of FS and PS rats were significantly increased relative to control group. ***p* < 0.01, different from the control (Student-Newman-Keuls test).


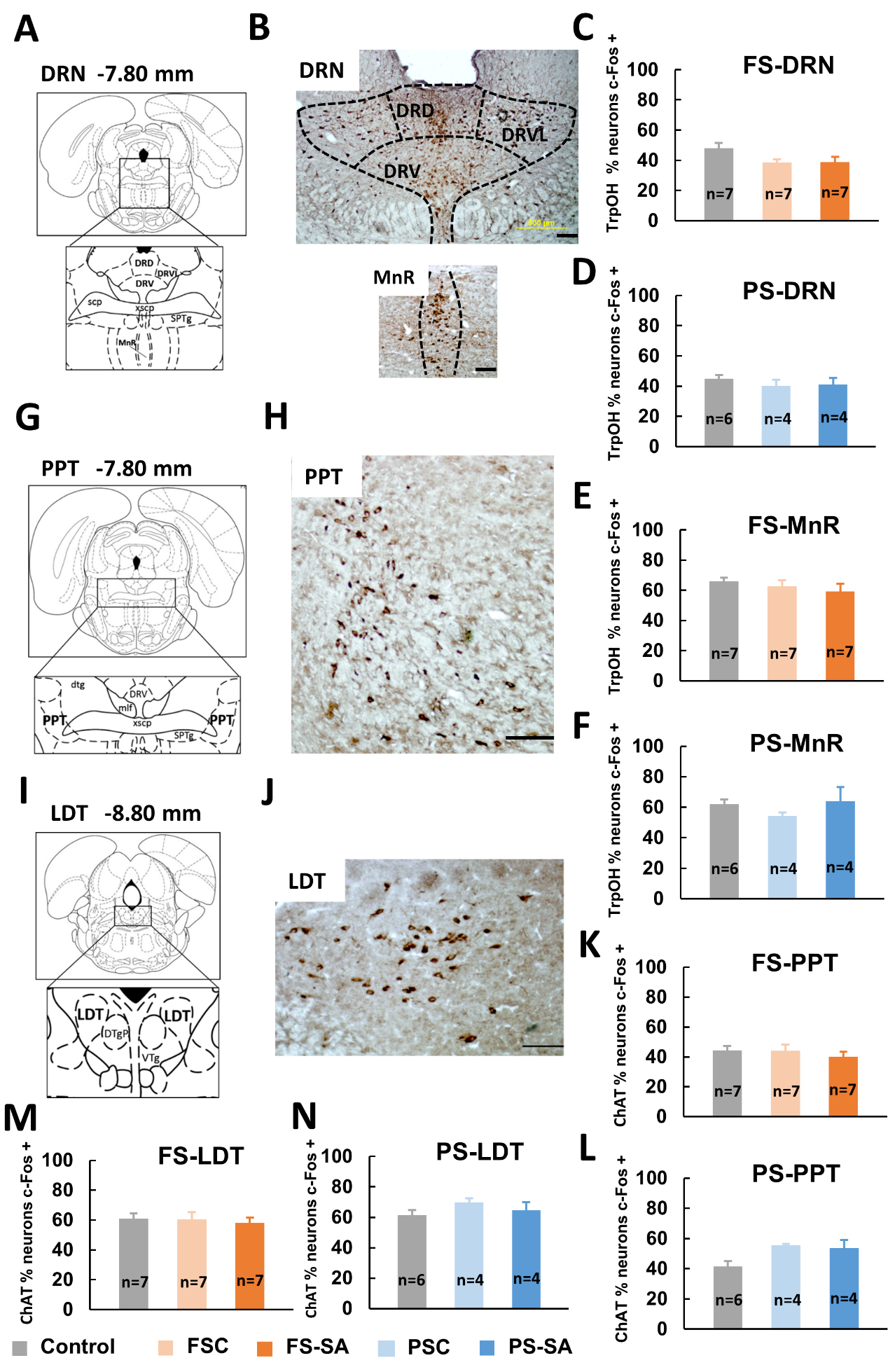


**Figure S2. Fos expression in TrpOH-immunostained neurons in the DRN/MnR and ChAT-immunostained neurons in the PPT/LDT.** **A.** Illustrations of brain sections of the DRN and MnR. **B.** Photomicrograph illustrating an example of Fos expression in TrpOH-immunostained neurons in the DRN and MnR. Scale bars = 100 μm. **C and D.** Fos expression positive ratio in serotonergic neurons in the DRN, there were no difference among groups. **E and F.** Fos expression positive ratio in serotonergic neurons in the MnR, there were no difference among groups. **G and I.** Illustrations of brain sections of the PPT and LDT. **H and J.** Photomicrograph illustrating an example of Fos expression in ChAT-immunostained neurons in the PPT and LDT. Scale bars = 100 μm. **K and L.** Fos expression positive ratio in cholinergic neurons in the PPT, there were no difference among groups. **M and N.** Fos expression positive ratio in cholinergic neurons in the LDT, there were no difference among groups. Data are represented as mean ± SEM.


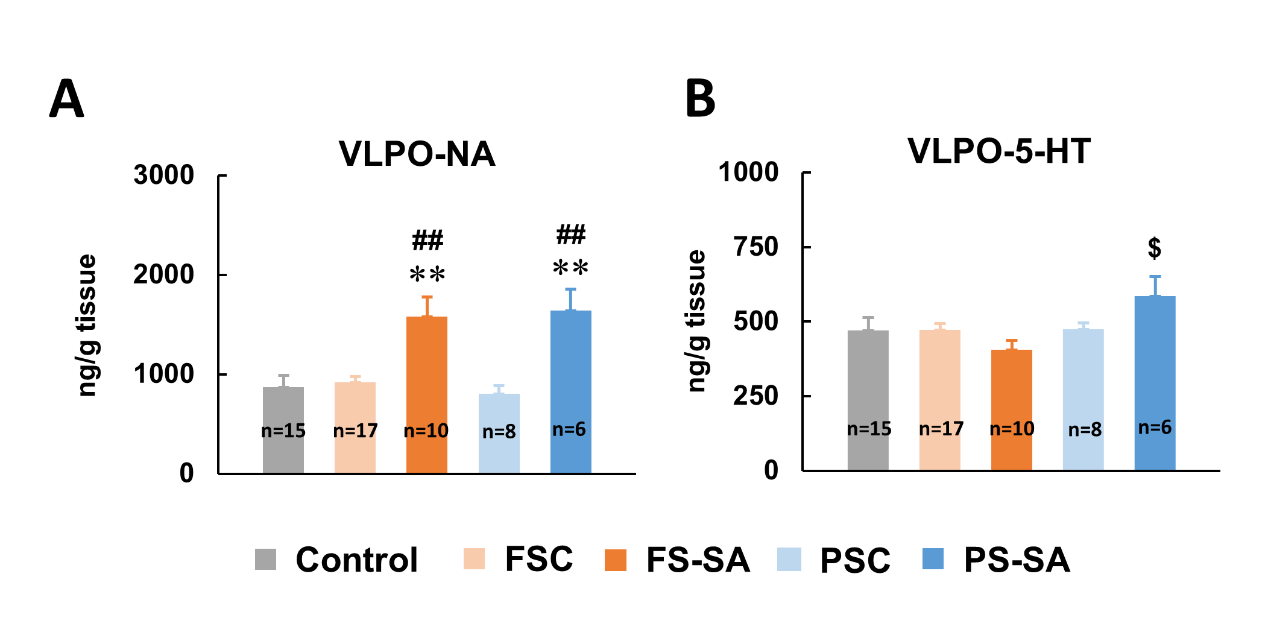


**Figure S3. Monoamine levels in sleep-promoting nucleus.** **A.** Norepinephrine levels significantly increased in the VLPO in both FS-SA and PS-SA groups. **B.** Compared to FS-SA group, 5-HT levels significantly increased in PS-SA rats. The data are expressed as mean ± SEM. **p* < 0.05, ***p* < 0.01, different from control group; ^#^*p* < 0.05, ^##^*p* < 0.01, different from the FSC/PSC group; ^$^*p* < 0.05, different from FS-SA group (Student-Newman-Keuls test). The data are expressed as mean ± SEM.


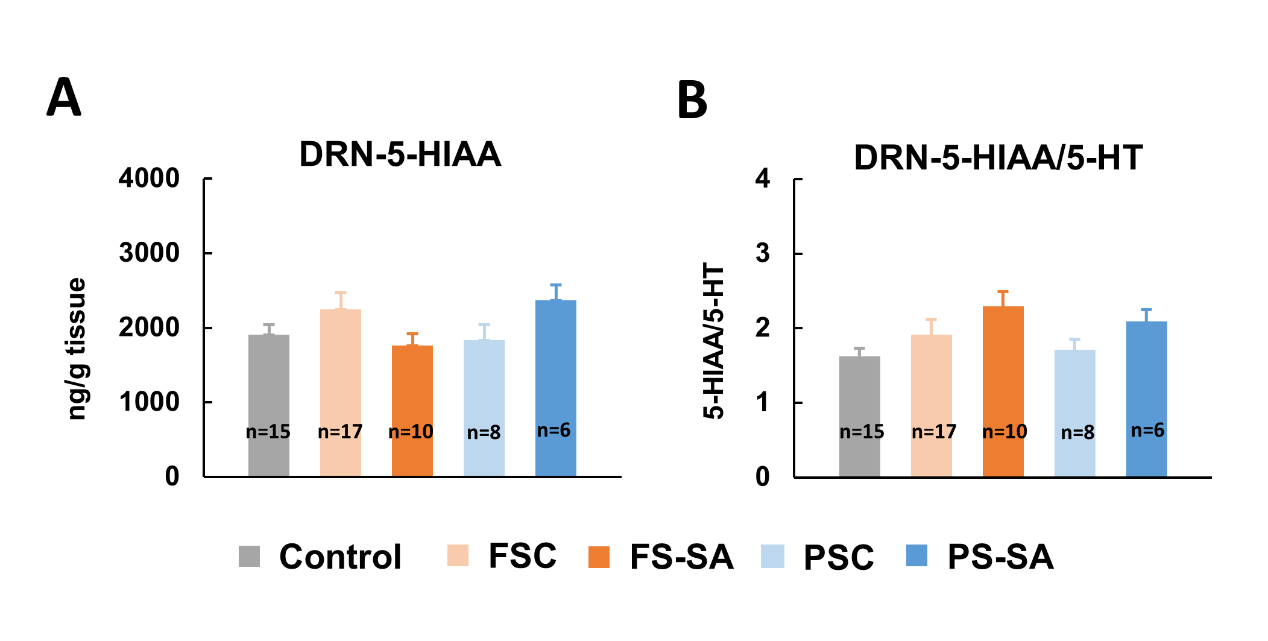


Figure S4. The metabolism level of 5-HT in DRN. A. 5- hydroxyindole acetic acid (5-HIAA) levels of FS-SA and PS-SA didn’t show significant differences in DRN (*F*_4, 51_ = 1.139, *p* = 0.349). B. 5-HIAA/5-HT ratio didn’t change significantly among groups (*F*_4, 51_ = 1.676, *p* = 0.170) (Student-Newman-Keuls test). The data are expressed as mean ± SEM.

**Supplemental Tables**

Table S1. The levels of monoamine neurotransmitters in arousal related nuclei.

| **Group**  **Brain region** | | | | | **NE**  **(ng/g)** | | **5-HT**  **(ng/g)** | | **5-HIAA**  **(ng/g)** | | **5-HIAA/**  **5-HT** | |  |
| --- | --- | --- | --- | --- | --- | --- | --- | --- | --- | --- | --- | --- | --- |
| LC | | Con | | | 1507.16 ± 131.00 | | 348.96 ± 34.84 | | 942.95 ± 153.04 | | 2.75 ± 0.35 | |  |
|  |  | FSC | | | 1659.60 ± 147.94 | | 413.86 ± 44.89 | | 1176.97 ± 158.16 | | 3.02 ± 0.27 | |  |
|  |  | FS-SA | | | 2289.27 ± 300.72 | | 514.01 ± 65.35 | | 1217.83 ± 122.16 | | 2.53 ± 0.32 | |  |
|  |  | PSC | | | 1256.24 ±123.93 | | 377.65 ± 63.88 | | 1313.20 ± 336.49 | | 3.64 ± 0.54 | |  |
|  |  | PS-SA | | | 2445.66 ± 366.92 | | 470.33 ± 120.62 | | 1032.96 ± 197.13 | | 2.36 ± 0.32 | |  |
| DRN | | Con | | | 1688.57 ± 127.25 | | 1201.72 ± 77.83 | | 1904.36 ± 134.38 | | 1.62 ± 0.11 | |  |
|  |  | FSC | | | 1335.99 ± 139.51 | | 1345.14 ± 159.50 | | 1798.07 ± 174.73 | | 1.91 ± 0.21 | |  |
|  |  | FS-SA | | | 1397.33 ± 233.03 | | 798.98 ± 99.37 | | 1758.20 ±219.09 | | 2.29 ± 0.20 | |  |
|  |  | PSC | | | 1338.41 ± 127.62 | | 1033.27 ± 112.55 | | 1833.86 ± 208.48 | | 1.78 ± 0.18 | |  |
|  |  | PS-SA | | | 1679.84 ± 382.77 | | 1764.23 ± 199.01 | | 2368.09 ± 391.75 | | 2.09 ± 0.36 | |  |
|  | VLPO | | | Con | | 868.00 ± 122.93 | | 469.83 ± 44.02 | | 1163.31 ± 147.59 | | 2.58 ± 0.32 | |
|  |  |  |  | FSC | | 917.39 ± 60.29 | | 472.46 ± 20.83 | | 1158.42 ± 49.91 | | 2.40 ± 0.21 | |
|  |  |  |  | FS-SA | | 1578.26 ± 199.64 | | 405.07 ± 31.57 | | 1191.58 ± 104.73 | | 2.85 ± 0.39 | |
|  |  |  |  | PSC | | 844.77 ± 79.80 | | 474.68 ± 27.19 | | 1220.80 ± 103.49 | | 2.62 ± 0.23 | |
|  |  |  |  | PS-SA | | 1640.81 ± 218.26 | | 584.92 ± 67.16 | | 1398.00 ± 77.54 | | 2.55 ± 0.33 | |
|  | Pef | | Con | | | 1236.87 ± 151.88 | | 580.85 ± 49.15 | | 1164.79 ± 117.04 | | 2.05 ± 0.17 | |
|  |  |  | FSC | | | 1297.73 ± 171.93 | | 619.90 ± 48.57 | | 1222.70 ± 81.80 | | 2.02 ± 0.14 | |
|  |  |  | FS-SA | | | 1239.57 ± 118.50 | | 556.05 ± 66.33 | | 1087.36 ± 82.30 | | 2.24 ± 0.28 | |
|  |  |  | PSC | | | 1273.28 ± 178.64 | | 632.43 ± 57.50 | | 1186.53 ± 140.49 | | 1.96 ± 0.20 | |
|  |  |  | PS-SA | | | 1028.41 ± 138.77 | | 575.32 ± 62.13 | | 1015.82 ± 118.79 | | 1.78 ± 0.18 | |

**Table S2. The incidence of startled awakening 21 days after stress exposure in different estrus phases** in preliminary experiments

| Estrus  phase  Group | Proestrus | Estrus | Metestrus | Diestrus |
| --- | --- | --- | --- | --- |
| FS-SA | 25.0% | 16.6% | 6.3% | 8.3% |
| PS-SA | 15.0% | 12.5% | 10.0% | 9.4% |

**Captions for Videos S1 to S3:**

Video S1: Normal awakening

Video S2: FS-SA rats came to “startle awake”

Video S3: PS-SA rats came to “startle awake”
